# The Obesity-Associated Polymorphisms *FTO* rs9939609 and *MC4R* rs17782313 and Endometrial Cancer Risk in Non-Hispanic White Women

**DOI:** 10.1371/journal.pone.0016756

**Published:** 2011-02-08

**Authors:** Galina Lurie, Mia M. Gaudet, Amanda B. Spurdle, Michael E. Carney, Lynne R. Wilkens, Hannah P. Yang, Noel S. Weiss, Penelope M. Webb, Pamela J. Thompson, Keith Terada, Veronica Wendy Setiawan, Timothy R. Rebbeck, Jennifer Prescott, Irene Orlow, Tracy O'Mara, Sara H. Olson, Steven A. Narod, Rayna K. Matsuno, Jolanta Lissowska, Xiaolin Liang, Douglas A. Levine, Loic Le Marchand, Laurence N. Kolonel, Brian E. Henderson, Montserrat Garcia-Closas, Jennifer Anne Doherty, Immaculata De Vivo, Chu Chen, Louise A. Brinton, Mohammad R. Akbari, Australian National Endometrial Cancer Study Group, Epidemiology of Endometrial Cancer Consortium (E2C2), Marc T. Goodman

**Affiliations:** 1 Cancer Epidemiology Program, Cancer Research Center of Hawaii, University of Hawaii, Honolulu, Hawaii, United States of America; 2 Epidemiology Research Program, American Cancer Society, Inc., Atlanta, Georgia, United States of America; 3 Queensland Institute of Medical Research, Brisbane, Post Office Royal Brisbane Hospital, Brisbane, Australia; 4 Department of Obstetrics and Gynecology, John A. Burns School of Medicine, University of Hawaii, Honolulu, Hawaii, United States of America; 5 Division of Cancer Epidemiology and Genetics, National Cancer Institute, National Institutes of Health, Department of Health and Human Services, Bethesda, Maryland, United States of America; 6 Department of Epidemiology, School of Public Health, University of Washington, Seattle, Washington, United States of America; 7 Program in Epidemiology, Division of Public Health Sciences, Fred Hutchinson Cancer Research Center, Seattle, Washington, United States of America; 8 Department of Preventive Medicine, Keck School of Medicine, University of Southern California, Los Angeles, California, United States of America; 9 University of Pennsylvania School of Medicine, Philadelphia, Pennsylvania, United States of America; 10 Abramson Cancer Center, Philadelphia, Pennsylvania, United States of America; 11 Department of Epidemiology, Program in Molecular and Genetic Epidemiology, Harvard School of Public Health, Boston, Massachusetts, United States of America; 12 Channing Laboratory, Department of Medicine, Brigham and Women's Hospital, Harvard Medical School, Boston, Massachusetts, United States of America; 13 Department of Epidemiology and Biostatistics, Memorial Sloan-Kettering Cancer Center, New York, New York, United States of America; 14 Hormone Dependent Cancer Group, Institute of Health and Biomedical Innovation, Queensland University of Technology, Brisbane, Australia; 15 Women's College Research Institute, University of Toronto, Toronto, Ontario, Canada; 16 Johns Hopkins Bloomberg School of Public Health, Baltimore, Maryland, United States of America; 17 Department of Cancer Epidemiology and Prevention, M. Sklodowska-Curie Memorial Cancer Center and Institute of Oncology, Warsaw, Poland; 18 Department of Surgery, Memorial Sloan-Kettering Cancer Center, New York, New York, United States of America; 19 Department of Otolaryngology: Head and Neck Surgery, School of Medicine, University of Washington, Seattle, Washington, United States of America; Ohio State University Medical Center, United States of America

## Abstract

Overweight and obesity are strongly associated with endometrial cancer. Several independent genome-wide association studies recently identified two common polymorphisms, *FTO* rs9939609 and *MC4R* rs17782313, that are linked to increased body weight and obesity. We examined the association of *FTO* rs9939609 and *MC4R* rs17782313 with endometrial cancer risk in a pooled analysis of nine case-control studies within the Epidemiology of Endometrial Cancer Consortium (E2C2). This analysis included 3601 non-Hispanic white women with histologically-confirmed endometrial carcinoma and 5275 frequency-matched controls. Unconditional logistic regression models were used to assess the relation of *FTO* rs9939609 and *MC4R* rs17782313 genotypes to the risk of endometrial cancer. Among control women, both the *FTO* rs9939609 *A* and *MC4R* rs17782313 *C* alleles were associated with a 16% increased risk of being overweight (p = 0.001 and p = 0.004, respectively). In case-control analyses, carriers of the *FTO* rs9939609 *AA* genotype were at increased risk of endometrial carcinoma compared to women with the *TT* genotype [odds ratio (OR)  = 1.17; 95% confidence interval (CI): 1.03–1.32, p = 0.01]. However, this association was no longer apparent after adjusting for body mass index (BMI), suggesting mediation of the gene-disease effect through body weight. The *MC4R* rs17782313 polymorphism was not related to endometrial cancer risk (per allele OR = 0.98; 95% CI: 0.91–1.06; p = 0.68). *FTO* rs9939609 is a susceptibility marker for white non-Hispanic women at higher risk of endometrial cancer. Although *FTO* rs9939609 alone might have limited clinical or public health significance for identifying women at high risk for endometrial cancer beyond that of excess body weight, further investigation of obesity-related genetic markers might help to identify the pathways that influence endometrial carcinogenesis.

## Introduction

Endometrial cancer is the most common invasive gynecologic cancer in U.S. women with an estimated 43,470 new cases expected in 2010 [1]. Obesity is a well established risk factor for endometrial cancer among both premenopausal and postmenopausal women [2]. Adult obesity is associated with a 2- to 5-fold increased risk for endometrial cancer and may account for 40% of endometrial cancer incidence [2], [3]. Etiologic models of endometrial carcinogenesis have focused primarily on the role of steroid hormones, especially the effect of a deficiency in progestagen relative to estrogen on endometrial cells [4], [5]. According to the ‘unopposed estrogen’ hypothesis, the mitogenic effects of estrogen on the endometrium, especially if not counterbalanced by progestagen, increase the risk of malignancy. Adipocytes are the primary source of estrogen in postmenopausal women when the ovarian production of estrogen has ceased [6]. Obesity in postmenopausal women enhances circulating levels of estrogen through increased production and aromatization of androstenedione in adipose tissue, as well as decreased production of sex-hormone-binding globulin and reduced 2-hydroxylation of estradiol [7]. Among premenopausal women, obesity is thought to contribute to endometrial cancer risk through an association with progesterone deficiency during the luteal phase of the menstrual cycle, resulting in cellular proliferation and reduced desquamation of the endometrium [5], [7].

Recently, several independent large-scale genome-wide association studies (GWAS) reported an association of *fat mass and obesity associated (FTO; MIM: 610966)* and *melanocortin-4 receptor (MC4R; MIM: 155541)* gene polymorphisms with obesity and BMI in Caucasian populations [8]–[12]. Associations of BMI with common variants in these two loci have been reproduced in multiple studies [13], [14]. Carriage of the *FTO* rs9939609 *A* and *MC4R* rs17782313 *C* alleles was estimated to increase the risk of obesity by 31% [8] and 12% [11], respectively.

The protein encoded by *FTO* has been described as a Fe(II)- and 2-oxoglutarate-dependent oxygenase that might operate as a DNA demethylase. The human *FTO* gene is expressed in many tissues including mesenteric fat, pancreas, liver and adipose tissue, with the highest concentrations found in the hypothalamus [8], [15]. Experimental animal studies provide direct functional evidence that *FTO* underlies obesity [16]. Two studies have demonstrated that *FTO* gene expression in the arcuate nucleus of the hypothalamus is regulated by fasting [17], [18], suggesting that FTO may be important to the control of energy homeostasis. The *MC4R* gene encodes the MC_4_ protein, a ubiquitously expressed G-protein-coupled receptor that binds α-melanocyte stimulating hormone (α-MSH) [19]. Experimental studies show that *MC4R* is a key regulator of energy balance, influencing food intake and energy expenditure through functionally divergent central melanocortin neuronal pathways [20].

To examine the relation between the obesity-associated *FTO* rs9939609 and *MC4R* rs17782313 and endometrial cancer risk, we utilized pooled data within the Epidemiology of Endometrial Cancer Consortium (E2C2) [21]. We also evaluated the association of these single nucleotide polymorphisms (SNPs) with the endometrioid histological type of endometrial carcinoma. Endometrioid carcinoma comprises approximately 80% of all sporadic endometrial cancers [22]. It is a prototypical estrogen-dependent tumor with a strong, definitive link to obesity. Thus, we hypothesized a stronger association of the *FTO* rs9939609 *A* allele and *MC4R* rs17782313 C allele and risk of the endometrioid type of endometrial carcinoma than with nonendometrioid types.

## Results

The *FTO* rs9939609 minor allele (*A*) frequency among pooled controls was 0.40 (range by study: 0.39 to 0.47) (**Table S1**). The *MC4R* rs17782313 minor allele (*C*) frequency among controls was 0.25 (range: 0.23 to 0.28).

The minor alleles for both *FTO* rs9939609 and *MC4R* rs17782313 were associated with a 16% per allele increased risk of being overweight (p = 0.001 and p = 0.004, respectively) (**Table 1**).

**Table 1:** Association of *FTO* rs9939609 and *MC4R* rs17782313 SNPs with BMI (kg/m^2^) in control women.

Genotype	All	Lean women (BMI <25 kg/m^2^)	Overweight women (BM I≥25 kg/m^2^)		
	N (%)	N (%)	N (%)	a OR (95% CI)	a *P*
*FTO* rs9939609	4291	2278	2013		
*TT*	1536 (36)	861 (38)	675 (33)	1.00 (reference)	
*TA*	2032 (47)	1069 (47)	963 (48)	1.11 (0.97–1.27)	*0.12*
*AA*	723 (17)	348 (15)	375 (19)	**1.37 (1.14–1.64)**	***0.003***
Per allele				**1.16 (1.06–1.27)**	***0.001***
*MC4R* rs17782313	3900	2128	1772		
*TT*	2231 (57)	1266 (59)	965 (54)	1.00 (reference)	
*TC*	1390 (36)	720 (34)	670 (38)	**1.22 (1.06–1.39)**	***0.005***
*CC*	279 (7)	142 (7)	137 (8)	1.26 (0.98–1.62)	*0.08*
Per allele				**1.16 (1.05–1.29)**	***0.004***

a Odds ratios (OR), 95% confidence intervals (CI), and pair-wise p-values (1 d.f.) adjusted for age and study.

Note: statistically significant associations (P<0.05) are presented in bold font.

**Figure 1:**
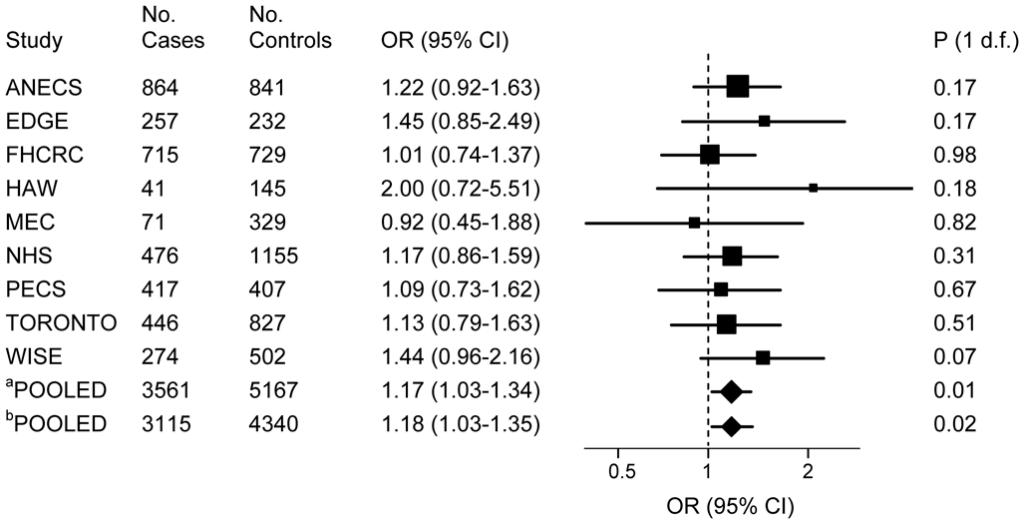
Association of the *FTO* rs9939609 with endometrial carcinoma risk in non-Hispanic white women. Forest plot of the ORs and 95% CIs comparing endometrial carcinoma risk for the *FTO* rs9939609 rare allele homozygotes (*AA* genotype) versus common allele homozygotes (*TT* genotype) for nine studies included in the pooled analysis. The pooled^a^ OR for all studies was 1.17 [95% CI: 1.03–1.34; p (1 d.f.)  = 0.01.] P for heterogeneity of effects by study = 0.87. The pooled^b^ OR for studies including incident cases only (excluding TORONTO study) was 1.18 [95% CI: 1.03–1.35; p (1 d.f.)  = 0.02]. P for heterogeneity of effects between studies with incident cases vs. prevalent cases (TORONTO)  = 0.78. Pooling was performed by combining all data using study as fixed and random effects (results were the same).

**Figure 2:**
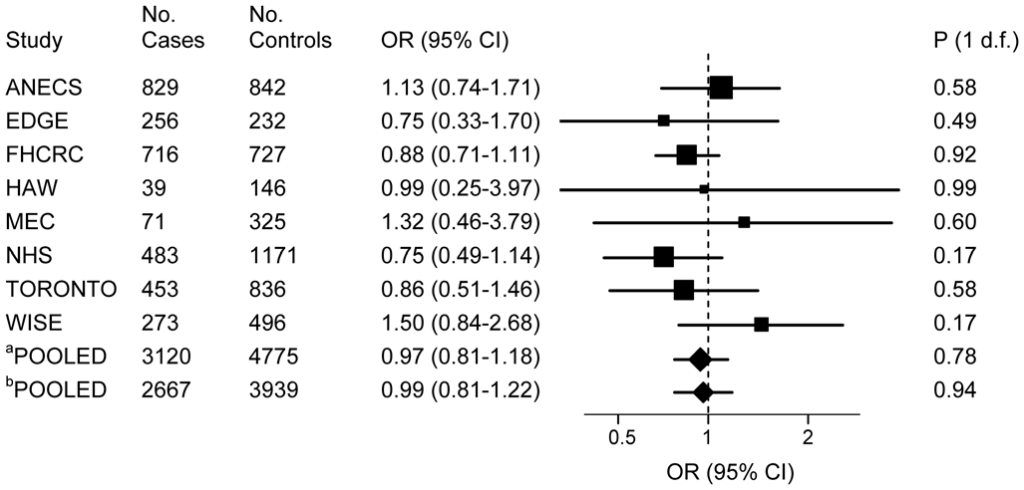
Association of the *MC4R* rs17782313 with endometrial carcinoma risk in non-Hispanic white women. Forest plot of the ORs and 95% CIs comparing endometrial carcinoma risk for the *MC4R* rs17782313 rare allele homozygotes (CC genotype) versus common allele homozygotes (*TT* genotype) for eight studies included in the pooled analysis. The pooled^a^ OR for all studies combined was 0.97 [95% CI: 0.81–1.18; p (1 d.f.)  = 0.78]. P for heterogeneity of effects by study = 0.49. The pooled^b^ OR for studies including incident cases only (excluding TORONTO study) was 0.99 [95% CI: 0.81–1.22; p (1 d.f.)  = 0.94]. P for heterogeneity of effects between studies with incident cases vs. prevalent cases (TORONTO)  = 0.68. Pooling was performed by combining all data using study as fixed and random effects (results were the same).

In the pooled analysis, the *FTO* rs9939609 *AA* genotype was associated with an increased risk of endometrial cancer (OR = 1.17; 95% CI: 1.03–1.32; p = 0.01) compared to women with the *TT* genotype (**Table 2**). No heterogeneity of the genotype associations with endometrial cancer was observed by study in any of the models (**Table S2 and** **Figure 1**). Excluding WISE study (with genotypes deviating from HWE) did not alter the association of SNPs with endometrial cancer risk (OR = 1.15; 95% CI: 1.01–1.32; p = 0.04). The *FTO* rs9939609 association with risk remained consistent in the analysis restricted to incident cases in which the TORONTO study participants were excluded (OR = 1.18; 95% CI: 1.03–1.35; p = 0.02). No heterogeneity of effects was observed between the TORONTO study and studies including incident cases only (p = 0.78). In the subset of women with BMI data available, the association of the *FTO* rs9939609 *AA* genotype with risk remained the same (**Table 3**). However, the association of the *FTO* rs9939609 *A* allele with risk was no longer observed after adjusting for BMI (**Table 3**) or in the analysis by BMI strata (**Table S3**). The majority of cases were diagnosed with endometrioid carcinomas (N = 1,419 cases; 63%). In the analyses restricted to the endometrioid histological subtype, the *FTO* rs9939609 *AA* versus *TT* genotype was slightly strengthened (OR = 1.26; 95% CI: 1.04–1.52; p = 0.02) (**Table 2**), but again completely attenuated after adjusting for BMI (**Table 3**). No associations of the *MC4R* polymorphism with endometrial cancer risk were found in any of the models (**Figure 2**, **Tables 2**, **3**, **S2**, **and S3**).

**Table 2:** Association of the *FTO* rs9939609 and *MC4R* rs17782313 with endometrial carcinoma risk.

Genotypes	Cases N (%)	Controls N (%)	a OR (95% CI)	a *P*
All women				
*FTO* rs9939609	3561	5167		
*TT*	1236 (35)	1856 (36)	1.00 (reference)	
*TA*	1662 (47)	2463 (48)	0.99 (0.91–1.10)	*0.99*
*AA*	663 (18)	848 (16)	1.17 (1.03–1.32)	*0.01*
Per allele			1.07 (1.01–1.14)	*0.04*
*MC4R* rs17782313	3120	4775		
*TT*	1814 (58)	2751 (58)	1.00 (reference)	
*TC*	1094 (35)	1693 (35)	0.98 (0.8*9*–1.08)	*0.71*
*CC*	212 (7)	331 (7)	0.97 (0.81–1.18)	*0.78*
Per allele			0.98 (0.91–1.06)	*0.68*
Cases with endometrioid carcinoma and controls from studies with available histology data				
*FTO* rs9939609	1403	2778		
*TT*	490 (35)	1025 (37)	1.00 (reference)	
*TA*	648 (46)	1298 (47)	1.04 (0.90–1.21)	*0.58*
*AA*	265 (19)	455 (16)	1.26 (1.04–1.52)	*0.02*
Per allele			1.11 (1.01–1.22)	*0.03*
*MC4R* rs17782313	1368	2768		
*TT*	799 (58)	1613 (58)	1.00 (reference)	
*TC*	488 (35)	974 (35)	1.01 (0.87–1.16)	*0.96*
*CC*	91 (7)	181 (7)	0.99 (0.76–1.32)	*0.99*
Per allele			1.01 (0.90–1.12)	*0.98*

a ORs, 95% CIs, and pair-wise p-values (1 d.f.) from the logistic regression models adjusted for age and study.

**Table 3:** Association of the *FTO* rs9939609 and *MC4R* rs17782313 with endometrial carcinoma risk among women with BMI data available.

	Cases N (%)	Controls N (%)	Before adjusting for BMI		After adjusting for BMI	
			a OR (95% CI)	a *P*	b OR (95% CI)	b *P*
All women						
*FTO* rs9939609	3061	4291				
*TT*	1063 (35)	1536 (36)	1.00 (reference)		1.00 (reference)	
*TA*	1415 (46)	2032 (47)	0.98 (0.88–1.09)	*0.72*	0.93 (0.84–1.04)	*0.23*
*AA*	583 (19)	723 (17)	1.17 (1.02–1.34)	***0.03***	1.04 (0.90–1.21)	*0.57*
Per allele			1.07 (0.99–1.14)	*0.07*	1.01 (0.94–1.08)	*0.84*
*MC4R* rs17782313	2619	3900				
*TT*	1517 (58)	2231 (57)	1.00 (reference)		1.00 (reference)	
*TC*	915 (35)	1390 (36)	0.98 (0.87–1.09)	*0.65*	0.90 (0.81–1.01)	*0.08*
*CC*	187 (7)	279 (7)	1.00 (0.82–1.23)	*0.99*	0.93 (0.75–1.14)	*0.47*
Per allele			0.99 (0.91–1.07)	*0.78*	0.94 (0.86–1.02)	*0.12*
Cases with endometrioid carcinoma and controls from studies with available histology data						
*FTO* rs9939609	1378	2753				
*TT*	481 (35)	1010 (37)	1.00 (reference)		1.00 (reference)	
*TA*	637 (46)	1289 (47)	1.03 (0.89–1.20)	*0.66*	1.01 (0.86–1.17)	*0.98*
*AA*	260 (19)	454 (16)	1.24 (1.02–1.50)	***0.03***	1.09 (0.89–1.34)	*0.40*
Per allele			1.10 (0.99–1.21)	*0.05*	1.04 (0.94–1.15)	*0.47*
*MC4R* rs17782313						
*TT*	1354	2743	1.00 (reference)		1.00 (reference)	
*TC*	785 (58)	1598 (58)	1.01 (0.87–1.16)	*0.98*	0.91 (0.78–1.06)	*0.23*
*CC*	479 (35)	966 (35)	1.01 (0.77–1.33)	*0.93*	0.93 (0.69–1.25)	*0.62*
Per allele	90 (7)	179 (7)	1.01 (0.90–1.12)	*0.94*	0.94 (0.84–1.05)	*0.28*

a ORs, 95% CIs, and pair-wise p-values (1 d.f.) from the logistic regression models adjusted for age and study.

b ORs, 95% CIs, and pair-wise p-values (1 d.f.) from the logistic regression models adjusted for age and study, and BMI (continuous variable).

## Discussion

In this pooled analysis of non-Hispanic white women from the United States, Poland, Canada and Australia, we found that carriers of the *FTO* rs9939609 *AA* genotype were at increased risk of endometrial carcinoma. This genetic association appears to be mediated through a relation of rs9939609 to a woman's weight, as no independent effect of this SNP was observed after accounting for BMI.

Experimental evidence suggests that obesity associated SNPs in intron 1 of the *FTO* gene are associated with altered gene expression [23]. Using primer extension analysis, Berulava et al. [23] determined the ratio of allelic *FTO* transcript levels in unspliced heterogeneous nuclear DNA preparations from blood and fibroblasts of individuals heterozygous for rs9939609. The *FTO* transcripts containing the *A* (‘risk’) allele were more abundant than those with T allele (mean 1.38; 95% CI: 1.31–1.44).

The *FTO* rs9939609 SNP is related to body weight through an influence on energy intake and satiety [18], [24]–[29]. The rs9939609 A allele was associated with increased energy intake in adults [25] and children [24], [26]–[28], [30] in several epidemiological studies. Den Hoed et al. [29] reported that women with *TA* and *AA* rs9939609 genotypes had significantly lower postprandial responses to hunger and satiety compared to *TT* carriers. Wardle et al. [24] observed that children with two copies of the lower-risk *FTO* alleles ate less than those with one or two higher-risk alleles and concluded that the T allele is protective against overeating by promoting responsiveness to internal signals of satiety. In addition, two studies reported an association of the rs9939609 *A* allele with decreased lipolysis [31], [32].

The lack of an independent effect of the *MC4R* rs17782313 SNP was unexpected and needs further investigation. Although the power of our *MC4R* analysis was modest, odds ratios were close to one, providing no suggestion of an association of this SNP with endometrial cancer risk among non-Hispanic white women. Further study of additional genetic correlates of body weight will assist in clarifying whether the *FTO* relation to endometrial cancer risk is unique among ‘obesity-associated’ genes.

A strength of this pooled analysis was the large sample size available within the E2C2. A large number of genetic variants and quantitative trait loci that potentially predispose to obesity have been reported, but only a few have been convincingly confirmed in multiple independent large scale investigations [33] and *FTO* remains the strongest genetic determinant of common obesity characterized to date. A limitation of this analysis was that histology was available for only 62% of women. Furthermore, we did not have detailed information on menopausal hormone use, weight at different periods in life, body fat distribution, or other factors that may influence endometrial cancer risk [3]. However, no association of *FTO* genotype with menopausal status or menopausal hormone use was observed in the subset of women for whom this information was available. Finally, the use of self-reported height and weight might have resulted in nondifferential misclassification and thus underestimation of the true effects.

Although important gaps exist in our understanding of the molecular pathways leading to increased weight and obesity, our data provide novel evidence that the *FTO* rs9939609 *AA* genotype is associated with endometrial cancer risk among non-Hispanic white women. As more common genetic variants associated with overweight and obesity are identified, these might help to identify the pathways that influence endometrial carcinogenesis.

## Methods

### Ethics statement

All participating studies were approved by the review boards and ethics committees of their parent institutions and participating hospitals, including Queensland Institute of Medical Research, Brisbane, Australia, for the Australian National Endometrial Cancer Study (ANECS); the Institutional Review Board (IRB) at Memorial Sloan-Kettering Cancer Center, NJ, USA, for the Estrogen, Diet, Genetics, and Endometrial Cancer (EDGE) study; the IRB of the Fred Hutchinson Cancer Research Center, WA, USA, for the Fred Hutchinson Cancer Research Center Case-Control Study (FHCRC); the IRB of the University of Hawaii, HI, USA, for the Hawaii Endometrial Cancer Study (HAW); the IRBs of the Universities of Hawaii and Southern California, for the Multiethnic Cohort Study (MEC); the Committee on Use of Human Subjects of the Brigham and Women's Hospital, MA, USA for the Nurses' Health Study (NHS); the National Cancer Institute Central IRB, Bethesda, MD, USA, the Ethical Committee of The Maria Sklodowska-Curie Memorial Cancer Center and Institute of Oncology (Warsaw, Poland), and the Bioethical Committee of the Nofer Institute of Occupational Medicine (Lodz, Poland) for the Polish Endometrial Cancer Study (PECS); the Research Ethics Board of the Women's College Research Institute, Toronto, ON, Canada, for the Toronto Case-Control Endometrial Cancer Study; the Committee on Studies Involving Human Subjects of the University of Pennsylvania, PA, USA, for the Women's Insights and Shared Experiences Study (WISE).

Written informed consent was obtained from all participants.

### Study Design and Population

Based on Epidemiology of Endometrial Cancer Consortium (E2C2) procedures, we submitted a formal proposal describing our hypothesis and methods to the steering committee and to all consortium members. Genotyping of the proposed SNPs was performed in the individual laboratories of investigators expressing an interest in collaboration, following a similar protocol. All data were combined in the E2C2 coordinating center. Nine studies participating in this pooled analysis (**Tables 4** **and S4**) included 3601 women with primary incident endometrial carcinoma and 5275 women who were free of endometrial cancer and did not have history of hysterectomy. Six studies were population-based case-control studies, two studies were case-control studies nested within a cohort, and one study was hospital-based. All studies except the TORONTO study included incident endometrial cancer cases exclusively. Epidemiological data were collected using structured questionnaires. All data were combined in the E2C2 coordinating center. Age at diagnosis for cases or age at interview for controls was available for all study participants. *FTO* rs9939609 genotype data were available for 8728 women (3561 cases and 5167 controls) and *MC4R* rs17782313 genotype data were available for 7895 women (3120 cases and 4775 controls). Self-reported BMI data were available for 7459 (84%) of women; data were missing for women from the Toronto study (n = 1313; 14.5%) and for 1.5% of women from other studies. Histology data were available for 2243 (62%) cases. Data on menopausal status were available for 907 cases and 885 controls (20%) and use of any menopausal hormones were available for 3050 cases and 3803 controls (77% of women).

**Table 4:** Description of the studies included in the pooled analysis of *FTO* rs9939609 and *MC4R* rs17782313 and endometrial carcinoma risk.

Study Name	Location	Study Design	Cases (N)	Mean age (SD), yrs	Controls (N)	Mean age (SD), yrs
ANECS (Australian National Endometrial Cancer Study)	Australia	Population-based case-control	877	62.0 (9.3)	860	56.3 (12.0)
EDGE (Estrogen, Diet, Genetics, and Endometrial Cancer)	New Jersey, USA	Population-based case-control	258	61.8 (9.3)	233	65.2 (9.9)
FHCRC (Fred Hutchinson Cancer Research Center Case-Control Study)	Washington, USA	Population-based case-control	719	59.7 (6.1)	730	59.2 (6.1)
HAW (Hawaii Endometrial Cancer Study)	Hawaii, USA	Population-based case-control	42	64.5 (10.3)	146	56.6 (11.2)
MEC (Multiethnic Cohort Study)	California and Hawaii, USA	Nested case-control	73	64.9 (8.2)	337	61.7 (8.8)
NHS (Nurses' Health Study)	11 US States	Nested case-control	484	62.8 (8.4)	1195	62.4 (8.2)
PECS (Polish Endometrial Cancer Study)	Lodz and Warsaw, Poland	Population-based case-control	417	60.8 (8.4)	407	60.9 (8.9)
TORONTO (Toronto Case-Control Endometrial Cancer Study)	Canada	Hospital-based case-control	454	60.7 (12.1)	859	56.2 (10.2)
WISE (Women's Insights and Shared Experiences)	Pennsylvania, USA	Population based case-control	277	63.0 (8.1)	508	62.0 (8.1)
POOLED			3601	61.5 (8.9)	5275	59.7 (9.7)

### Genotyping

Genotyping was performed in the individual laboratories using 5′ nuclease TaqMan allelic discrimination assay (TaqMan, Applied Biosystems) following the same protocol. We used the following criteria to measure the acceptability of the genotyping results: (1) inclusion of ≥3% sample duplicates, (2) concordance rate for duplicate samples ≥99%, (3) overall call rate by study ≥95% and (4) intermixing of cases and controls on each plate. All studies met these criteria. Genotyping quality was also assessed using tests for Hardy-Weinberg equilibrium (HWE). The genotype distribution for both SNPs among controls was consistent with HWE in all but one study (WISE, p = 0.01) for rs9939609 and one study (NHS, p = 0.02) for rs17782313. Exclusion of these studies did not appreciably affect the reported results. *MC4R* rs17782313 genotype data were not available for the PEC study (417 cases and 407 controls).

### Statistical analysis

All analyses were completed in the SAS statistical software package version 9.2 (SAS Institute Inc., Cary, NC). Fisher's goodness-of-fit test was used to assess whether allele frequency distributions among controls were consistent with HWE. Unconditional multiple logistic regression models were used to calculate odds ratios (ORs) and 95% confidence intervals (CIs) for the association of genotype with endometrial cancer risk and BMI, calculated as the ratio of weight in kilograms divided by the square of height in meters. BMI was used as continuous variable, as well as categorical with two levels: lean women (BMI <25 kg/m^2^) and overweight women (BMI ≥25 kg/m^2^). The genotype for each SNP was treated as a non-ordered categorical variable to test for heterogeneity and as an ordered categorical variable (with three levels: 0, 1, 2; one assigned to each genotype) to test for an allele-dose effect. Homozygous carriers of the common *FTO* rs9939609 and *MC4R* rs17782313 *T* alleles were used as the reference group for these models. Heterogeneity of effects by study was examined using two different methods. First, we included study site as a fixed effect covariate and evaluated heterogeneity of the association of genotypes with risk by study, using a Wald test of the genotype-study interaction term. Second, we included study site as a random effect using SAS GLIMMIX procedure (the results were the same). To evaluate potential confounders, the distributions of genotypes among controls were examined by factors associated with ovarian cancer risk (age, menopausal status, and use of menopausal hormones) (**Table S5**). Age (continuous variable) was included in all models to account for residual confounding by imperfect matching. A Wald test was used to compare the associations of genotypes with endometrial cancer risk by study and BMI strata. Power calculations were performed using QUANTO software (http:hydra.usc.edu/gxe) and were based on population incidence rates of endometrial cancer of 24.4 per 100,000 women per year. These rates are based on cases diagnosed in 2001–2005 from 17 Surveillance Epidemiology and End Results (SEER) geographic areas [1]. Calculated minimal detectable ORs (MDOR) are presented in **Table S6**.

## Supporting Information

Table S1 *FTO* rs9939609 and *MC4R* rs17782313 genotype frequencies in white non-Hispanic women by study and overall.
(DOC)

Table S2 Association of the *FTO* rs9939609 and *MC4R* rs17782313 SNPs with endometrial carcinoma risk among non-Hispanic white women by study.
(DOC)

Table S3 Association of *FTO* rs9939609 and *MC4R* rs17782313 with endometrial carcinoma risk among white-non-Hispanic women by BMI strata.
(DOC)

Table S4 Case ascertainment and selection of controls.
(DOC)

Table S5 Frequency distribution of age, menopausal status, and menopausal hormone use by *FTO* rs9939609 and *MC4R* rs17782313 genotypes.
(DOC)

Table S6 Minimal detectable ORs (MDORs) for *FTO* rs9939606 and *MC4R* rs17782313 at power 80%, type I error = 0.05.
(DOC)
