# Supplementary material for: The Obesity-Associated Polymorphisms FTO rs9939609 and MC4R rs17782313 and Endometrial Cancer Risk in Non-Hispanic White Women
Source: PLoS One. 2011 Feb 8;6(2):e16756. doi: 10.1371/journal.pone.0016756 (PMC3035652; doi:10.1371/journal.pone.0016756)
Supplement: Table S1 — FTO rs9939609 and MC4R rs17782313 genotype frequencies in white non-Hispanic women by study and overall. (DOC) [file pone.0016756.s001.doc]

Table S1. *FTO* rs9939609 and *MC4R* rs17782313 genotype frequencies in white non-Hispanic women by study and overall

| Study Name | Cases (N) | Controls (N) | *FTO* rs9939609 genotype | | | | | | MAF among controls | ^a^ *P* |
| --- | --- | --- | --- | --- | --- | --- | --- | --- | --- | --- |
|  |  |  | N (%) Cases | | | N (%) Controls | | |  |  |
|  |  |  | *TT* | *TA* | *AA* | *TT* | *TA* | *AA* |  |  |
| ANECS | 864 | 841 | 320 (37) | 382 (44) | 162 (19) | 310 (37) | 398 (47) | 133 (16) | 0.39 | *0.78* |
| EDGE | 257 | 232 | 80 (31) | 121 (47) | 56 (22) | 71 (31) | 126 (54) | 35 (15) | 0.42 | *0.09* |
| FHCRC | 715 | 729 | 264 (37) | 343 (48) | 108 (15) | 279 (38) | 335 (46) | 115 (16) | 0.39 | *0.39* |
| HAW | 41 | 145 | 14 (34) | 17 (42) | 10 (24) | 53 (36) | 72 (50) | 20 (14) | 0.39 | *0.57* |
| MEC | 71 | 329 | 30 (42) | 27 (38) | 14 (20) | 117 (36) | 153 (46) | 59 (18) | 0.41 | *0.47* |
| NHS | 476 | 1155 | 179 (38) | 205 (43) | 92 (19) | 425 (37) | 543 (47) | 187 (16) | 0.40 | *0.54* |
| PECS | 417 | 407 | 105 (25) | 222 (53) | 90 (22) | 112 (27) | 207 (51) | 88 (22) | 0.47 | *0.68* |
| TORONTO | 446 | 827 | 152 (34) | 226 (51) | 68 (15) | 294 (36) | 415 (50) | 118 (14) | 0.39 | *0.14* |
| WISE | 274 | 502 | 92 (34) | 119 (43) | 63 (23) | 195 (39) | 214 (43) | 93 (18) | 0.40 | *0.01* |
| Pooled | 3561 | 5167 | 1236 (35) | 1662 (47) | 663 (18) | 1856 (36) | 2463 (48) | 848 (16) | 0.40 |  |
|  |  |  | *MC4R* rs17782313 genotype | | | | | | MAF among controls | ^a^ *P* |
|  |  |  | No. (%) Cases | | | No. (%) Controls | | |  |  |
|  |  |  | *TT* | *TC* | *CC* | *TT* | *TC* | *CC* |  |  |
| ANECS | 829 | 842 | 457 (55) | 316 (38) | 56 (7) | 499 (59) | 294 (36) | 49 (6) | 0.23 | *0.51* |
| EDGE | 256 | 232 | 164 (64) | 80 (31) | 12 (5) | 133 (57) | 85 (37) | 14 (6) | 0.24 | *0.93* |
| FHCRC | 716 | 727 | 424 (59) | 235 (33) | 57 (8) | 412 (57) | 259 (35) | 56 (8) | 0.26 | *0.09* |
| HAW | 39 | 146 | 24 (61) | 12 (31) | 3 (8) | 75 (51) | 59 (41) | 12 (8) | 0.28 | *0.93* |
| MEC | 71 | 325 | 38 (54) | 28 (39) | 5 (7) | 197 (61) | 108 (33) | 20 (6) | 0.23 | *0.32* |
| NHS | 483 | 1171 | 283 (59) | 167 (34) | 33 (7) | 643 (55) | 428 (37) | 100 (8) | 0.27 | *0.02* |
| TORONTO | 453 | 836 | 267 (59) | 163 (36) | 23 (5) | 495 (59) | 291 (35) | 50 (6) | 0.23 | *0.41* |
| WISE | 273 | 496 | 157 (58) | 93 (34) | 23 (8) | 297 (60) | 169 (34) | 30 (6) | 0.23 | *0.37* |
| Pooled | 3120 | 4775 | 1814 (58) | 1094 (35) | 212 (7) | 2751 (58) | 1693 (35) | 331 (7) | 0.25 |  |

Abbreviations: MAF, minor allele frequency.

^a^ P from the Fisher’s goodness of fit test assessing deviation of genotype frequencies among controls from those expected under Hardy-Weinberg equilibrium.

Note: *MC4R* rs17782313 genotype data were not available for the PEC study.
