# Supplementary material for: The Obesity-Associated Polymorphisms FTO rs9939609 and MC4R rs17782313 and Endometrial Cancer Risk in Non-Hispanic White Women
Source: PLoS One. 2011 Feb 8;6(2):e16756. doi: 10.1371/journal.pone.0016756 (PMC3035652; doi:10.1371/journal.pone.0016756)
Supplement: Table S2 — Association of the FTO rs9939609 and MC4R rs17782313 SNPs with endometrial carcinoma risk among non-Hispanic white women by study. (DOC) [file pone.0016756.s002.doc]

Table S2. Association of the *FTO* rs9939609 and *MC4R* rs17782313 SNPs with endometrial carcinoma risk among non-Hispanic white women by study.

| Study | Cases (N) | Controls N) | Heterozygotes and rare allele homozygotes | | | Log-additive model | |
| --- | --- | --- | --- | --- | --- | --- | --- |
|  |  |  | *FTO* rs9939609 | | | | |
|  |  |  | *TA* | *AA* | ^a^ P (2 d.f.) | Per allele | ^a^ *P* for trend |
|  |  |  | ^a^ OR (95% CI) | ^a^ OR (95% CI) |  | ^a^ OR (95% CI) |  |
| ANECS | 864 | 841 | 0.95 (0.77-1.19) | 1.22 (0.92-1.63) | *0.22* | 1.07 (0.94-1.24) | *0.29* |
| EDGE | 257 | 232 | 0.87 (0.58-1.31) | 1.45 (0.85-2.49) | *0.13* | 1.15 (0.89-1.50) | *0.28* |
| FHCRC | 715 | 729 | 1.08 (0.86-1.36) | 1.01 (0.74-1.37) | *0.76* | 1.02 (0.88-1.18) | *0.81* |
| HAW | 41 | 145 | 1.10 (0.48-2.53) | 2.00 (0.72-5.51) | *0.37* | 1.37 (0.82-2.29) | *0.23* |
| MEC | 71 | 329 | 0.72 (0.40-1.28) | 0.92 (0.45-1.88) | *0.51* | 0.91 (0.64-1.31) | *0.62* |
| NHS | 476 | 1155 | 0.90 (0.71-1.14) | 1.17 (0.86-1.59) | *0.20* | 1.05 (0.90-1.22) | *0.54* |
| PECS | 417 | 407 | 1.14 (0.83-1.59) | 1.09 (0.73-1.62) | *0.72* | 1.05 (0.86-1.28) | *0.63* |
| TORONTO | 446 | 827 | 1.08 (0.83-1.40) | 1.13 (0.79-1.63) | *0.77* | 1.07 (0.90-1.27) | *0.47* |
| WISE | 274 | 502 | 1.16 (0.83-1.63) | 1.44 (0.96-2.16) | *0.21* | 1.20 (0.98-1.46) | *0.08* |
| Pooled | 3561 | 5167 | 0.99 (0.91-1.10) | 1.17 (1.03-1.34) | *0.02* | 1.07 (1.01-1.14) | *0.04* |
| ^b^ *P* |  |  |  | *0.73* |  | *0.92* |  |
|  |  |  | *MC4R* rs17782313 | | | | |
|  |  |  | *CT* | *CC* | P (2 d.f.) | Per allele | ^a^ P for trend |
|  |  |  | ^a^ OR (95% CI)* | ^a^ OR (95% CI)* |  | ^a^ OR (95% CI)* |  |
| ANECS | 829 | 842 | 1.18 (0.95-1.45) | 1.13 (0.74-1.71) | *0.30* | 1.12 (0.95-1.32) | *0.18* |
| EDGE | 256 | 232 | 0.75 (0.51-1.11) | 0.75 (0.33-1.70) | *0.32* | 0.80 (0.59-1.39) | *0.16* |
| FHCRC | 716 | 727 | 0.88 (0.71-1.11) | 0.98 (0.66-1.45) | *0.55* | 0.94 (0.80-1.11) | *0.49* |
| HAW | 39 | 146 | 0.72 (0.32-1.61) | 0.99 (0.25-3.97) | *0.71* | 0.86 (0.47-1.52) | *0.63* |
| MEC | 71 | 325 | 1.38 (0.80-2.40) | 1.32 (0.46-3.79) | *0.49* | 1.25 (0.83-1.89) | *0.28* |
| NHS | 483 | 1171 | 0.89 (0.71-1.11) | 0.75 (0.49-1.14) | *0.30* | 0.88 (0.74-1.04) | *0.12* |
| TORONTO | 453 | 836 | 1.01 (0.79-1.29) | 0.86 (0.51-1.46) | *0.85* | 0.97 (0.80-1.18) | *0.77* |
| WISE | 273 | 496 | 1.03 (075-1.41) | 1.50 (0.84-2.68) | *0.39* | 1.13 (0.89-1.43) | *0.31* |
| Pooled | 3120 | 4775 | 0.98 (0.8*9-*1.08) | 0.97 (0.81-1.18) | *0.91* | 0.98 (0.91-1.06) | *0.68* |
| ^b^ *P* |  |  |  | *0.49* |  | *0.17* |  |

^a^ ORs, 95% CIs, and p-values from the logistic regression models adjusted for age and, in combined analyses, by study.

^b^ P for heterogeneity of the association of the SNPs with risk by study was estimated using a Wald test of the genotype-study interaction term.
