# Supplementary material for: The Obesity-Associated Polymorphisms FTO rs9939609 and MC4R rs17782313 and Endometrial Cancer Risk in Non-Hispanic White Women
Source: PLoS One. 2011 Feb 8;6(2):e16756. doi: 10.1371/journal.pone.0016756 (PMC3035652; doi:10.1371/journal.pone.0016756)
Supplement: Table S3 — Association of FTO rs9939609 and MC4R rs17782313 with endometrial carcinoma risk among white-non-Hispanic women by BMI strata. (DOC) [file pone.0016756.s003.doc]

Table S3. Association of *FTO* rs9939609 and *MC4R* rs17782313 with endometrial carcinoma risk among white-non-Hispanic women by BMI strata.

| Genotype | Lean women (BMI <25 kg/m^2^) | | | | Overweight and obese women (BMI ≥ 25kg/m^2^ ) | | | |
| --- | --- | --- | --- | --- | --- | --- | --- | --- |
|  | N=3310 | | | | N=4149 | | | |
|  | Cases N (%) | Controls N (%) | ^a^ OR (95% CI) | ^a^ *P* | Cases N (%) | Controls N (%) | ^a^ OR (95% CI) | ^a^ *P* |
| *FTO* rs9939609 |  |  |  |  |  |  |  |  |
| N | 990 | 2278 |  |  | 2071 | 2013 |  |  |
| *TT* | 387 (39) | 861 (38) | 1.00 (reference) |  | 676 (33) | 675 (33) | 1.00 (reference) |  |
| *TA* | 440 (44) | 1069 (47) | 0.89 (0.75-1.05) | *0.17* | 975 (47) | 963 (48) | 0.97 (0.84-1.13) | *0.68* |
| *AA* | 163 (17) | 348 (15) | 1.05 (0.84-1.32) | *0.67* | 420 (20) | 375 (19) | 1.05 (0.87-1.27) | *0.62* |
| Per allele | |  | 1.00 (0.89-1.11) | *0.93* |  |  | 1.02 (0.93-1.12) | *0.73* |
| ^b^ *P* | |  |  |  |  |  |  | *0.67* |
| *MC4R rs17782313* |  |  |  |  |  |  |  |  |
| N | 872 | 2128 |  |  | 1747 | 1772 |  |  |
| *TT* | 540 (62) | 1266 (59) |  |  | 977 (56) | 965 (54) | 1.00 (reference) |  |
| *TC* | 277 (32) | 720 (34) | 0.90 (0.76-1.07) | *0.24* | 638 (36) | 670 (38) | 0.92 (0.70-1.21) | *0.23* |
| *CC* | 55 (6) | 142 (7) | 0.92 (0.66-1.29) | *0.64* | 132 (8) | 137 (8) | 0.91 (0.78-1.06) | *0.54* |
| Per allele | |  | 0.93 (0.82-1.06) | *0.29* |  |  | 0.94 (0.84-1.05) | *0.25* |
| ^b^ *P* | |  |  |  |  |  |  | *0.98* |

^a^ ORs, 95% CIs, and p-values estimated using unconditional logistic regression adjusted for age, study, and BMI (continuous variable).

^b^ *P* for heterogeneity of the association of the SNPs with risk by BMI category was estimated using a Wald test of the genotype-BMI category interaction term.
