# Supplementary material for: The Obesity-Associated Polymorphisms FTO rs9939609 and MC4R rs17782313 and Endometrial Cancer Risk in Non-Hispanic White Women
Source: PLoS One. 2011 Feb 8;6(2):e16756. doi: 10.1371/journal.pone.0016756 (PMC3035652; doi:10.1371/journal.pone.0016756)
Supplement: Table S4 — Case ascertainment and selection of controls. (DOC) [file pone.0016756.s004.doc]

Table S4. Case ascertainment and selection of controls

| Study Name | Case ascertainment | Selection of controls |
| --- | --- | --- |
| ANECS | Incident cases (diagnosed 2005-2007) ascertained through treatment centers and cancer registries (national coverage) | (1) Randomly selected from the national electoral roll (enrolment to vote is compulsory); (2) blood donors |
| EDGE | Incident cases (diagnosed 2001-2005) ascertained through New Jersey Cancer Registry | (1) Random digit dialing for women <65; (2) Medicare & Medicaid Services lists for women aged >65; (3) Area sampling for women aged >55 |
| FHCRC | Incident cases (diagnosed 1994-1995, 1997-1999, and 2003-2005) ascertained through the Cancer Surveillance System cancer registry affiliated with SEER | Randomly selected controls using random-digit dialing and Health Care Financing Administration files; frequency-matched to cases by age, county of residence, and reference year |
| HAW | Incident cases (diagnosed 1993-2007) ascertained through Hawaii Tumor Registry | Randomly selected from the participants in the annual survey of representative households that is conducted under statutory provision |
| MEC | Incident cases (diagnosed 1993-2003) ascertained through California and Hawaii tumor registries | Control participants were a subsample of controls randomly selected to serve as controls for a breast cancer case-control study nested within the MEC; had an intact uterus at baseline and remained free of breast and uterine cancer through December 31, 2003 |
| NHS | Incident cases diagnosed after cohort inception up to June 1, 2004 ascertained by self reports and confirmed by medical record review | Randomly selected from the cohort up to the time the case was diagnosed, matched by age, menopausal status, menopausal hormone use, and biospecimen type |
| PECS | Incident cases (diagnosed 2001-2003), ascertained through hospitals and cancer registry | Randomly selected from a database of all residents; frequency-matched by age and city |
| TORONTO | Cases have been enrolled from patients diagnosed with endometrial cancer in Toronto hospitals between 1996 and 2008 | Controls (free of cancer) had attended a screening clinic for healthy women at Women's College Hospital between 1999 and 2003 |
| WISE | Population-based ascertainment of incident cases was undertaken in nine counties in the Philadelphia metropolitan area between 1999-2002 | Controls were ascertained by random digit dialing from the same nine counties as the cases, and frequency matched on age and race |
