# Supplementary material for: The Obesity-Associated Polymorphisms FTO rs9939609 and MC4R rs17782313 and Endometrial Cancer Risk in Non-Hispanic White Women
Source: PLoS One. 2011 Feb 8;6(2):e16756. doi: 10.1371/journal.pone.0016756 (PMC3035652; doi:10.1371/journal.pone.0016756)
Supplement: Table S5 — Frequency distribution of age, menopausal status, and menopausal hormone use by FTO rs9939609 and MC4R rs17782313 genotypes. (DOC) [file pone.0016756.s005.doc]

Table S5. Frequency distribution of age, menopausal status, and menopausal hormone use by *FTO* rs9939609 and *MC4R* rs17782313 genotypes

|  | *FTO* | | | | | | | |
| --- | --- | --- | --- | --- | --- | --- | --- | --- |
|  | Controls | | | | Cases | | | |
|  | *TT* | *TA* | *AA* | ^a^ P | *TT* | *TA* | *AA* | ^a^ P |
| Age at diagnosis (yrs) | 59.9 (0.2) | 59.6 (0.2) | 59.7 (0.3) | 0.65 | 61.7 (0.3) | 61.4 (0.2) | 61.3 (0.3) | 0.54 |
| Menopausal status |  |  |  |  |  |  |  |  |
| N (%) premenopausal | 46 (14) | 54 (13) | 17 (12) |  | 14 (5) | 36 (9) | 13 (8) |  |
| N (%) postmenopausal | 273 (86) | 357 (87) | 130 (88) | 0.69 | 286 (95) | 386 (91) | 160 (92) | 0.13 |
| Use of menopausal hormones |  |  |  |  |  |  |  |  |
| N (%) used | 648 (47) | 832 (48) | 295 (48) |  | 507 (47) | 621 (45) | 222 (41) |  |
| N (%) never used | 725 (53) | 913 (52) | 315 (52) | 0.89 | 580 (53) | 757 (55) | 325 (59) | 0.07 |
|  | *MC4R* | | | | | | | |
|  | Controls |  |  |  | Cases |  |  |  |
|  | *TT* | *TC* | *CC* |  | *TT* | *TC* | *CC* |  |
| Age at diagnosis (yrs) | 59.4 (0.2) | 59.8 (0.2) | 60.0 (0.5) | 0.31 | 61.7 (0.2) | 61.3 (0.3) | 62.2 (0.6) | 0.25 |
| Menopausal status |  |  |  |  |  |  |  |  |
| N (%) premenopausal | 59 (12) | 50 (16) | 8 (14) |  | 31 (6) | 25 (8) | 7 (12) |  |
| N (%) postmenopausal | 445 (88) | 262 (84) | 48(86) | 0.21 | 501 (94) | 282 (92) | 52 (86) | 0.14 |
| Use of menopausal hormones |  |  |  |  |  |  |  |  |
| N (%) used | 1015 (48) | 621 (47) | 141 (53) |  | 823 (47) | 427 (41) | 94 (45) |  |
| N (%) never used | 1119 (52) | 714 (53) | 126 (47) | 0.17 | 913 (53) | 617 (59) | 114 (55) | 0.004 |

^a^ P values from general linear models comparing age (continuous variable) in subgroups by genotype and from chi-square test comparing frequency distribution of categorical variables (menopausal status and menopausal hormone use) by genotype.

Note: Age was available for all women. Menopausal status was available for 907 cases and 885 controls (20%) and use of any menopausal hormones was available for 3050 cases and 3803 controls (77% of women).
