# Supplementary material for: The Obesity-Associated Polymorphisms FTO rs9939609 and MC4R rs17782313 and Endometrial Cancer Risk in Non-Hispanic White Women
Source: PLoS One. 2011 Feb 8;6(2):e16756. doi: 10.1371/journal.pone.0016756 (PMC3035652; doi:10.1371/journal.pone.0016756)
Supplement: Table S6 — Minimal detectable ORs (MDORs) for FTO rs9939606 and MC4R rs17782313 at power 80%, type I error = 0.05. (DOC) [file pone.0016756.s006.doc]

Table S6. Minimal detectable ORs (MDORs) for *FTO* rs9939606 and *MC4R* rs17782313

at power 80%, type I error=0.05

| SNP | Disease incidence per 100,000 | Case (N) | Controls (N) | Control/case ratio | Minor allele frequency | MDOR |
| --- | --- | --- | --- | --- | --- | --- |
|  |  |  |  |  |  | Log-additive model |
|  | *Including all cases* | | | | | |
| *FTO* rs9939606 | 24.4 | 3561 | 5167 | 1.45 | 0.40 | 1.09 |
| *MC4R* rs17782313 | 24.4 | 3120 | 4775 | 1.53 | 0.25 | 1.11 |
|  | *Including endometrioid cases only* | | | | | |
| *FTO* rs9939606 | 24.4 | 1403 | 2778 | 1.98 | 0.40 | 1.14 |
| *MC4R* rs17782313 | 24.4 | 1368 | 2768 | 2.02 | 0.25 | 1.16 |
